# Concordance of acquired mutations between metastatic lesions and liquid biopsy in metastatic colorectal cancer

**DOI:** 10.2144/fsoa-2021-0059

**Published:** 2021-11-05

**Authors:** Fumitaka Taniguchi, Akihiro Nyuya, Toshiaki Toshima, Kazuya Yasui, Yoshiko Mori, Makoto Okawaki, Hiroyuki Kishimoto, Yuzo Umeda, Toshiyoshi Fujiwara, Hiroaki Tanioka, Yoshiyuki Yamaguchi, Ajay Goel, Takeshi Nagasaka

**Affiliations:** 1 Department of Gastroenterological Surgery, Okayama University Graduate School of Medicine, Dentistry & Pharmaceutical Sciences, Okayama, 700-8558, Japan; 2 Department of Clinical Oncology, Kawasaki Medical School, Kurashiki, 701-0192, Japan; 3 Department of Clinical Genetics & Digestive Tract & General Surgery, Saitama Medical Center, Saitama Medical University, Kawagoe, Saitama, 350-8550, Japan; 4 Department of Molecular Diagnostics & Experimental Therapeutics, Beckman Research Institute of City of Hope Comprehensive Cancer Center, CA 91016, USA

**Keywords:** Acquired mutations, *BRAF*, colorectal cancer, liquid biopsy, PCR-rSSO, *RAS*

## Abstract

**Aim:**

To evaluate whether PCR-reverse sequence-specific oligonucleotide can examine the concordance between liquid biopsy and metastatic lesions with acquired resistance.

**Materials & methods:**

We examined acquired mutations in chemoresistant lesions and blood obtained from four patients with *RAS* wild-type metastatic colorectal cancer who underwent treatment with anti-epidermal growth factor receptor antibodies.

**Results:**

In one patient, metastatic lesions harbored diverse acquired mutations in *KRAS* in all seven metastases; the two acquired mutations were detectable in blood collected after the patient acquired resistance. None of the other patients exhibited liquid biopsy mutations, except one, with a BRAF mutation confirmed in primary tumor and peritoneal dissemination.

**Conclusion:**

Liquid biopsy based on PCR-reverse sequence-specific oligonucleotide is a successful procedure for capturing acquired mutations with precise information on the *RAS* mutational spectrum.

Acquired resistance and primary resistance play major roles in anticancer treatment [1–3]. The epidermal growth factor receptor (EGFR)-targeted antibodies cetuximab and panitumumab can treat metastatic colorectal cancer (mCRC) negative for mutations in *KRAS* and *NRAS* exons 2–4 [4–6]. Although patients with metastatic CRC without activated *RAS* mutations show a clinical response to anti-EGFR antibodies, acquired resistance may develop. Indeed, several studies have identified acquired genetic alterations, including *KRAS, HER2* or *MET* amplification, and *KRAS, NRAS, BRAF* or *EGFR* mutations [1,3,7–12].

Recent studies have suggested characterizing genomic alterations in solid tumors by analyzing circulating tumor DNA (ctDNA) released from cancer cells into the plasma [13]. Currently, OncoBEAM-based liquid biopsy is a standard procedure for detecting *RAS* mutations in plasma [14,15]. Indeed, a previous study using OncoBEAM technology demonstrated that the mutant allele frequency (MAF) is as low as 0.1% of the acquired *RAS* mutant alleles [1,16]. However, approximately 10–18% of patients harbored *RAS* mutations in tissue that could not be detected in plasma [14,15]. The reason for this discordance may be attributed to tumor heterogeneity, lower circulating tumor DNA shedding, or lower tumor burden. Clinically, patients with mCRC at advanced stages possess multiple metastatic lesions in multiple organs. Therefore, in such cases, the heterogeneity of *RAS* mutations must be considered. However, a limited number of studies have evaluated the concordance of *RAS* mutational status in all metastatic lesions located in multiple organs and ctDNAs.

More importantly, although the OncoBEAM-based liquid biopsy has higher sensitivity for *RAS* mutant alleles and can demonstrate which exon is mutated, it cannot provide a precise *RAS* mutational spectrum, for example, G12C or G12A. *KRAS* mutations are often associated with resistance to targeted therapies and poor outcomes in patients with cancer; however, no selective *KRAS* inhibitor has yet been approved despite more than three decades of scientific effort [17–23]. Recent advances have led to the development of a small molecule, sotorasib, which specifically and irreversibly inhibits *KRAS* G12C through a unique interaction with a pocket of the switch II region. Sotorasib has demonstrated encouraging anticancer activity in patients with heavily pretreated advanced solid tumors harboring *KRAS* G12C mutations [24–27].

Thus, in the clinical setting, the RAS mutational spectrum identification will continue to improve as its role in multiple cancers is further recognized. Besides OncoBEAM technology, a PCR-reverse sequence-specific oligonucleotide (PCR-rSSO) method for detecting *RAS* mutations is now used in the clinical setting to detect *RAS* mutations in tumor tissues. The PCR-rSSO approach has a lower sensitivity for minor mutant alleles (the PCR-rSSO can detect >1% MAF) than OncoBEAM technology, but it can simultaneously identify all RAS mutational spectra. In this study, the PCR-rSSO method was examined for its concordance for identifying *RAS* mutations present in multiple metastatic lesions in multiple organs and ctDNAs in plasma throughout anti-EGFR therapy in patients with mCRC [15].

## Patients & methods

### Tumor samples

Four patients with mCRC who had primary tumors without activated *RAS* mutations were analyzed in this study. The patients were treated between 2011 and 2017 at the Okayama University Hospital, Japan. Each patient enrolled as a research subject in clinical trials (University Hospital Medical Information Network Center; IDs: 8377, 9698 and 11954).

**Figure 1.**
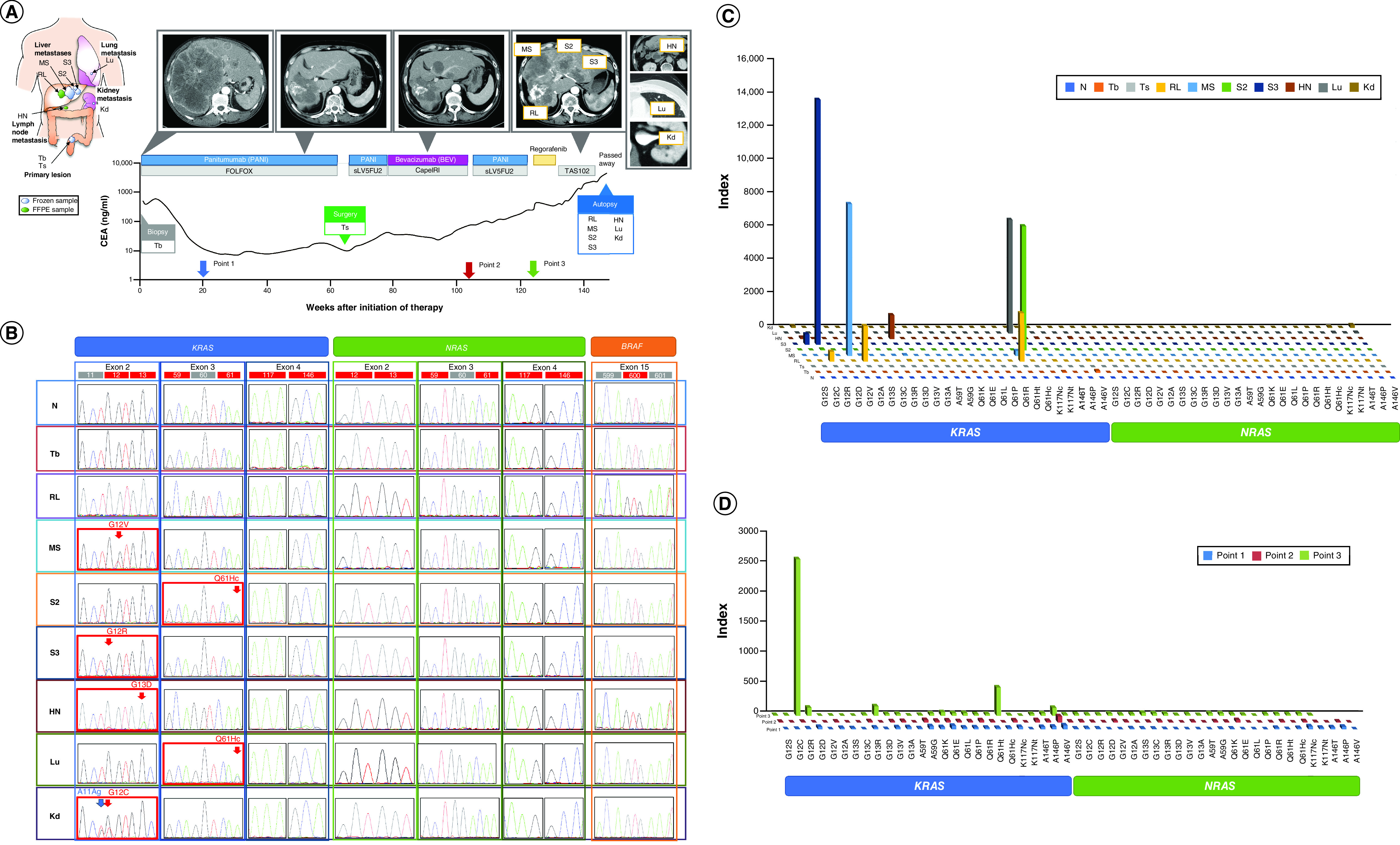
Summary of a patient with rectosigmoid cancer (patient 1) with unresectable multiple liver metastases. **(A)** Timeline of the treatment course of patient 1. The line graph abscissa indicates time, and the ordinate indicates CEA levels, scaled logarithmically. The computed tomography scan of the liver metastases (above) and chemotherapy regimen (below) are described for each time point. Blood was collected before the administration of chemotherapy at time points 1, 2 and 3 after the initiation of first-line chemotherapy. **(B)** Status of *RAS* and *BRAF* mutational metastases by Sanger sequencing. Mutational status of *KRAS, NRAS*, and *BRAF.* Sanger sequencing results are described for all samples, including primary lesions with distant focal metastasis. Numbers in red and gray boxes indicate the codon numbers, codons in red boxes indicate the hot spots and red arrows indicate the observed missense mutations. **(C)** Status of *RAS* mutational metastases by PCR-rSSO. Panels show index values of 12 types of *RAS* exon 2 (G12S, G12C, G12R, G12D, G12V, G12A, G13S, G13C, G13R, G13D, G13V and G13A), eight types of *RAS* exon 3 (A59T, A59G, Q61K, Q61E, Q61L, Q61P, Q61R and Q61H), and four types of *RAS* exon 4 (K117N, A146T, A146P and A146V) mutations by PCR-rSSO indices of metastatic specimens in the patient. **(D)** Detection of circulating *RAS* mutant DNA. The detection of circulating *RAS* mutant alleles in plasma by PCR-rSSO. The *KRAS* G12R mutant allele index value increased to 2600 in plasma obtained at time point 3. The index values of the *KRAS* G13D and Q61Hc mutant alleles also increased to 159 and 471, respectively, in plasma obtained at time point 3. CEA: Carcinoembryonic antigen; PCR-rSSO: Polymerase chain reaction-reverse sequence-specific oligonucleotide.

Patient 1’s primary tumors were obtained through biopsy before initiating any treatment (Tb) and by surgical resection before acquiring resistance during anti-EGFR treatment (Ts). Metastatic lesions were excised at morbid autopsy and included those of the liver (middle segment [MS], S2 and S3), hepatic lymph node (HN), lung (Lu) and kidney (Kd) (Figure 1A).

**Figure 2.**
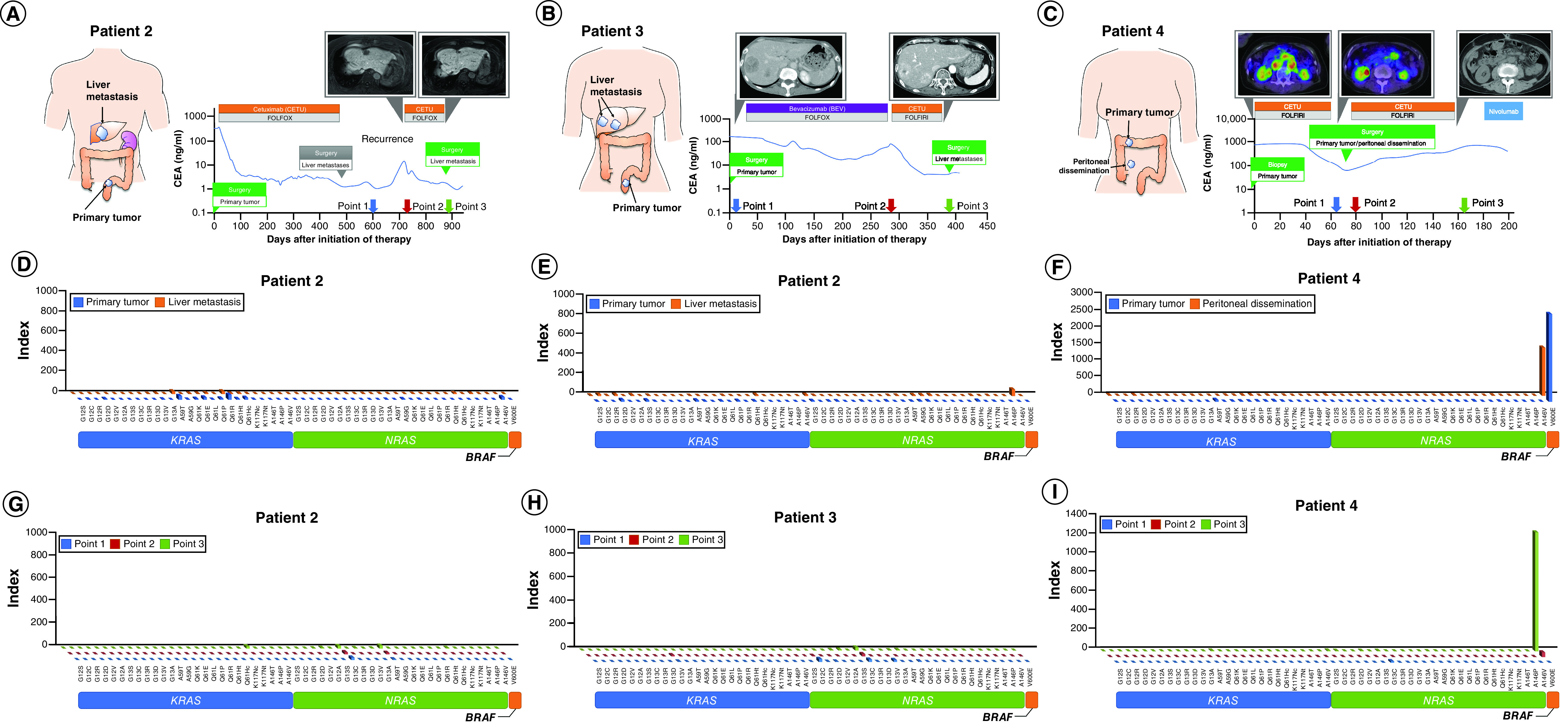
Summary of the three metastatic colorectal cancer patients. **(A–C)** Timeline of the treatment course of patients 2, 3 and 4. The line graph abscissa indicates time, and the ordinate indicates CEA levels, scaled logarithmically. Magnetic resonance imaging (patient 2, **A**), computed tomography (patient 3, **B**), and positron emission tomography (patient 4, **C**) scan of the liver metastases or peritoneal dissemination are described for each time point. Blood was collected at time points 1, 2 and 3. **(D–F)** *RAS* and *BRAF* mutational status in primary tumor and metastases by PCR-rSSO. Panels show the index values of 12 types of *RAS* exon 2 (G12S, G12C, G12R, G12D, G12V, G12A, G13S, G13C, G13R, G13D, G13V and G13A), eight types of *RAS* exon 3 (A59T, A59G, Q61K, Q61E, Q61L, Q61P, Q61R and Q61H), and four types of *RAS* exon 4 (K117N, A146T, A146P and A146V) mutations by PCR-rSSO indices of the primary tumor and metastatic specimens in the patients. **(G–I)** Detection of Circulating *RAS* and *BRAF* mutant DNA. The detection of circulating *RAS* and *BRAF* mutant alleles in plasma by PCR-rSSO. Patients 2 **(G)** and 3 **(H)** showed no mutation in liquid biopsy. In patient 4 **(I)**, the *BRAF* V600E mutant allele index value increased to 1242 in plasma obtained at time point 3. CEA: Carcinoembryonic antigen; PCR-rSSO: Polymerase chain reaction-reverse sequence-specific oligonucleotide.

The primary tumors from patient 2 (Figure 2A) and patient 3 (Figure 2D) were obtained by surgical resection before chemotherapy. A metastatic liver lesion from patient 2 was obtained after subsequent liver resection, and one from patient 3 was obtained at the initial liver resection.

The primary tumor of patient 4 was obtained by biopsy before initiating any treatment. A metastatic lesion of peritoneal dissemination was obtained by surgical resection after anti-EGFR treatment (Figure 2G).

Tissues obtained from autopsy or surgical resection were immediately stored at -80°C.

### Blood samples

Blood samples from patient 1 were collected before the development of progressive disease (PD) during first-line chemotherapy (time point 1) and after the patient acquired resistance to second-and third-line chemotherapies (time points 2 and 3, Figure 1A). The blood sample at time point 1 was collected from patient 2 after surgical resection of liver metastases at the right lobe. Blood samples from time points 2 and 3 were collected after confirmed recurrence in the liver and after a second resection of liver metastasis (Figure 2A). The time point 1 blood sample was collected from patient 3 before initiating treatment. The time point 2 blood sample was collected after PD development during bevacizumab (an antivascular endothelial growth factor antibody)-based chemotherapy. The time point 3 blood sample was collected after cetuximab treatment with tumor shrinkage (Figure 2D). In patient 4, the time point 1 blood sample was collected before PD development, the time point 2 blood sample was collected after surgical resection, and the time point 3 blood sample was collected after the patient acquired resistance to cetuximab treatment (Figure 2G). Plasma was separated immediately and stored at -80°C.

### Extraction of genomic DNA

Genomic DNA was extracted from fresh-frozen samples using the QIAamp DNA Mini Kit (Qiagen NV, Hilden, The Netherlands). Tumor DNA of several metastatic lesions was microscopically extracted from formalin-fixed paraffin-embedded (FFPE) specimens, which included metastases located in the liver (RL) and HN. DNA derived from FFPE specimens was extracted using the QIAamp DNA FFPE Tissue Kit (Qiagen NV). Circulating cell-free DNA was extracted from 200 μl of plasma using the QIAamp Blood Kit (Qiagen) per to the manufacturer’s instructions.

### Conventional sequencing

Sanger sequencing was performed to confirm mutations in *KRAS, NRAS* exons 2–4, and *BRAF* exon 15 (including codon 600) in all samples. The primer sequences for *KRAS, NRAS* exons 2–4, and *BRAF* exon 15, and the PCR conditions are described in Supplementary Table 1. The PCR products were purified using the QIAquick PCR Purification Kit (Qiagen) and were directly sequenced using the ABI PRISM^®^ 3100-Avant and SeqStudio Genetic Analyzer (Thermo Fisher Scientific, OH, USA).

### PCR-rSSO

Extensive *RAS* mutations (both *KRAS* and *NRAS* mutations) of DNA purified from FFPE, fresh-frozen tissues, and plasma were evaluated using the MEBGEN™ RASKET or RASKET-B KIT based on the Luminex^®^ technology (MBL, Nagoya, Japan). The assay was performed according to the manufacturer’s protocol. The MEBGEN RASKET KIT can simultaneously examine 12 types of *RAS* exon 2 (G12S, G12C, G12R, G12D, G12V, G12A, G13S, G13C, G13R, G13D, G13V and G13A), eight types of *RAS* exon 3 (A59T, A59G, Q61K, Q61E, Q61L, Q61P, Q61R and Q61H), and four types of *RAS* exon 4 (K117N, A146T, A146P and A146V) mutations [16]. The MEBGEN RASKET-B KIT was used to examine 12 types of *RAS* exon 2, eight types of *RAS* exon 3, four types of *RAS* exon 4 and the *BRAF* V600E mutation.

## Results

### Identification of acquired *RAS* mutations by conventional Sanger sequencing

In patient 1, there was no activating *RAS* mutation in the primary tumor biopsy specimen obtained before treatment, in the primary tumor tissue obtained after 1 year of panitumumab administration, or in a metastatic tumor in the right liver lobe (RL) that continuously responded to panitumumab with first-line systemic chemotherapy. The tumor shrank in response to a third-line panitumumab rechallenge (Figure 1A). *KRAS* sequences in metastatic specimens obtained during autopsy revealed diverse acquired mutations at different metastatic sites, indicating resistance to systemic chemotherapy, including anti-EGFR antibody treatment. Sanger sequencing in liver segments II (S2), III (S3), and MS detected acquired activating *KRAS* mutations resulting in G61Hc, G12R and G12V, respectively. The *KRAS* G12C, G13D and G61Hc mutations were also detected in metastases in the left kidney (Kd), a HN, and the left lung (Lu), respectively. No samples from primary and metastatic lesions harbored mutations in *NRAS* or *BRAF* (Figure 1B).

### Identification of acquired *RAS* mutations by a PCR-rSSO method

Sanger sequencing detected acquired mutations in six (87%) of the seven metastatic lesions in patient 1. Given the lower sensitivity for detecting mutant alleles by Sanger sequencing (∼20%), we could not exclude the possibility of associated metastatic lesions caused by low numbers of resistant cells already existing in the primary lesion.

We further analyzed all samples using the PCR-rSSO method, Rasket, which has high sensitivity in detecting extended *RAS* mutant alleles at lower frequencies (1–5%). A sample was mutation-positive when the index was estimated over the cut-off value and the sensitivity for each mutant allele was 1–5% [16].

There were no *KRAS* or *NRAS* mutations with indices higher than the cut-off values in DNA purified from pretreated primary tumor cells (Figure 1C & Supplementary Table 2). Additionally, PCR-rSSO analysis revealed the same mutation spectrum as Sanger sequencing in the patient except for the Kd and RL metastatic lesion. The index of the *KRAS* G12C allele captured from the Kd by PCR-rSSO analysis was 114, in other words, lower than the conventional cut-off value (index value: 300). This low index may have been influenced by the immediate synonymous mutation of A11Ag demonstrated by Sanger sequencing, as previously reported (Figure 1A) [16]. Interestingly, the metastatic tumor in the RL, which consistently responded to panitumumab and showed no mutation by Sanger sequencing, revealed multiple *KRAS* mutations, Q61Ht, G12A and G12R, with indices higher than the cut-off values. Although these mutant alleles were not sufficiently frequent to be detected by Sanger sequencing, heterogeneity in *RAS* mutant cancer cells may exist in the RL tumor mass.

In summary, by Sanger sequencing and PCR-rSSO, the seven metastatic lesions were found to harbor diverse acquired mutations in the *KRAS* gene: Q61Ht, G12A and G12R in RL; G12V in MS; Q61Hc in S2; G12R in S3; G12C in Kd; G13D in HN; and Q61Hc in Lu.

### Detection of acquired mutations in circulating cell-free DNA

We investigated whether acquired *RAS* mutations could be detected in the plasma samples using PCR-rSSO (Figure 1D). None of the seven diverse acquired mutations found in the resistant tumors were detectable before or after PD during the initial course of panitumumab treatment (time points 1 and 2 in Figure 1A & Supplementary Table 3), but two of the seven diverse acquired mutations were detectable at a significant level following PD confirmation during third-line panitumumab rechallenge (time point 3). The *KRAS* G12R and Q61Hc frequency confirmed in the resistant tumor in S3 and S2, respectively, were strikingly elevated in circulating cell-free DNA in a plasma sample collected at time point 3. None of the acquired mutations in the time point 3 plasma sample were detectable in the plasma of time points 1 and 2.

### Detection of acquired mutations in circulating cell-free DNA in patients 2, 3 & 4

We next evaluated the three patients with mCRC patients without activating *RAS* mutations. Patient 2 was initially resected for the primary tumor and then treated with FOLFOX plus cetuximab. After 1.5 years of chemotherapy administration, metastatic tumors in the RL that had continuously responded were resected (Figure 2A). Following liver resection, patient 2 was carefully followed without chemotherapy. However, the patient experienced a recurrence of single metastasis at the MS of the liver. FOLFOX plus cetuximab was re-introduced, which led to effective shrinkage of the metastatic lesion. Thus, second-time liver resection was performed. Neither the primary tumors obtained before treatment initiation nor the metastatic liver lesion after the second-time liver resection showed activated *RAS* mutations by the PCR-rSSO method (Figure 2B). Importantly, as patient 2 never experienced acquired resistance during the treatment course, the liquid biopsies among the three-time points showed no activating *RAS* mutations (Figure 2C).

Patient 3 was initially resected for the primary tumor and then treated with FOLFOX plus bevacizumab. Ten months after treatment, this first-line systemic chemotherapy failed, and FOLFIRI plus cetuximab was selected as the second-line treatment. The metastatic tumors in the RL responded rapidly to cetuximab, and liver resection was performed. Neither the primary tumor obtained before the initiation of treatment nor a metastatic liver lesion obtained after response to cetuximab showed activating *RAS* mutations by the PCR-rSSO method (Figure 2E). Similar to patient 2, patient 3 never experienced acquired resistance to cetuximab; therefore, it is reasonable that liquid biopsies among the three-time points showed no activating *RAS* mutations (Figure 2F).

Patient 4 was initially diagnosed with a primary tumor that had the *BRAF* V600E mutation and underwent treatment with FOLFIRI plus cetuximab as the first-line chemotherapy (Figure 2G & H). The primary tumor and peritoneal dissemination responded rapidly to cetuximab. After tumor shrinkage, the primary tumor and peritoneal dissemination were resected. After surgery, FOLFIRI plus cetuximab was continuously administered. Approximately 3 months after surgery, residual metastatic lesions grew, indicating resistance to systemic chemotherapy. Liquid biopsies at time points 1 and 2 were collected when the tumors responded rapidly to cetuximab. However, the liquid biopsy at time point 3 was collected after confirmation of acquired resistance to cetuximab. No *BRAF* V600E mutation nor *RAS* mutations were confirmed in plasma DNA (time points 1 and 2) while the tumors responded to cetuximab. In contrast, the *BRAF* V600E mutation was detectable in the plasma DNA collected after the patient became refractory to cetuximab (time point 3, Figure 2I).

## Discussion

Sanger sequencing is less sensitive for detecting minor cancer cell populations. In the current study, we aimed to determine the efficacy of a PCR-rSSO liquid biopsy method. Initially, we examined whether acquired mutations were detectable in metastatic sites collected after an autopsy by Sanger sequencing, which was confirmed by our results. Therefore, this study showed, for the first time, that acquired mutations can be detected in liquid biopsy by PCR-rSSO.

PD is generally determined by radiological evaluation. Liquid biopsy could be useful for early identification of individuals at risk of developing drug resistance before radiographic documentation of PD, as well as individuals who already have certain mutations in tumor burden [1,13–15]. Here, PCR-rSSO analysis was conducted on serial plasma samples and all metastatic lesions in multiple organs from patients who were treated with chemotherapies, including panitumumab and cetuximab.

In patient 1, none of the seven acquired mutations found in the resistant tumors were detectable before or after PD during the initial panitumumab treatment course (time points 1 and 2). However, three (G12R, G13D, and Q61Hc) of the seven acquired mutations (G12C, G12R, G12V, G12A, G13D, Q61Ht and Q61Hc) were detectable after PD was confirmed during third-line panitumumab rechallenge (time point 3). Indeed, two of the three acquired *RAS* mutations (G12R and Q61Hc) confirmed in the PCR-rSSO were beyond the cut-off values estimated by tumor tissue analysis. Of note, although the metastatic lesion at the RL was confirmed to possess three acquired mutations (G12R, G12A and Q61Ht) at autopsy, this lesion demonstrated an effective response to chemotherapies, including anti-EGFR antibody rechallenge.

In support of the results obtained from patient 1, among the liquid biopsies obtained from patient 4, only the time point 3 blood sample, collected after confirmation of radiological PD, demonstrated the *BRAF* V600E mutation that the primary tumor and peritoneal dissemination possessed. In contrast, as all blood specimens of patients 2 and 3 were collected when chemotherapies effectively caused tumor regression, these specimens did not show any activated mutations causing acquired resistance.

A previous study detected acquired mutation in plasma as early as 10 months before radiological PD [1]. Therefore, we also attempted to confirm whether acquired *RAS* variants were detectable in plasma before radiological PD. Unfortunately, given the conventional cut-off value of the PCR-rSSO, we could not detect acquired mutations before radiological PD. Our inability to detect ctDNA in the plasma samples might be partially explained by the differences in analytical technologies. The PCR-rSSO method used in this study can detect MAFs >1%, whereas the previous study with OncoBEAMing technology considered 0.1% MAFs mutation-positive [1,16]. The optimal cut-off value of OncoBEAMing is still debatable. Moreover, the PERSEIDA study reported an association between cut-off values of MAF of *RAS* mutant alleles in patients treated with panitumumab and their overall response rate [28]. If the cut-off value increases the sensitivity for lower MAF of *RAS* mutant alleles, the overall response rate in patients diagnosed as *RAS* wild-type was increased. However, similarly, the number of patients diagnosed with *RAS* mutant also showed an increased overall response rate to panitumumab treatment. Thus, patients who respond to anti-EGFR antibody treatment may be excluded to increase the sensitivity for lower MAF of *RAS* mutant alleles.

Indeed, the OncoBEAM-based liquid biopsy has a high sensitivity for *RAS* mutant alleles and can identify which exon is mutated. Still, it cannot provide a precise *RAS* mutational spectrum, for example, G12C or G12A. In contrast, although the PCR-rSSO strategy had less sensitivity for MAF, and even though the index values are semi-quantitative, the advantage of the PCR-rSSO is that it confirms all *RAS* mutations simultaneously. Thus, the PCR-rSSO may represent a useful and complementary tool for liquid biopsy to detect *RAS* mutations.

Even if we use the highest sensitivity method to detect mutant alleles of resistance in liquid biopsy, we missed the mutant alleles from the blood in 10–18% of patients with *RAS* mutations in tumor tissue [14,15]. This is likely due to tumor heterogeneity, lower ctDNA shedding, or lower tumor burden. In particular, the location of metastatic tumors may be important for detecting mutant alleles. Discrepancies of mutations between tumor tissue and ctDNA in patients with lung-only metastases occurred, but a higher agreement in liver metastases was found [15,29,30]. In line with this, our results from patient 1 also showed that the degree of *RAS* mutational concordance varied according to the metastatic site, e.g., *KRAS* G12R detected in ctDNA was confirmed in resistant tumors in S3, which appeared to be more aggressively progressed by radiographic findings.

At least two explanations could account for the development of acquired mutations [3,9]. First, resistant cells harboring these acquired mutations may be present in low numbers upon treatment initiation. Second, cells may have acquired a *de novo* activating mutation in response to the continued molecularly-targeted therapy. In the first model, the metastatic CRC response to EGFR-targeted therapies accompanies a selection of pre-existing resistant clones metastasized to the initial metastatic lesion. Therefore, if the acquired *RAS* mutations in this study were present at treatment initiation but at a low enough frequency to be undetectable by the two different procedures, at least some resistant metastatic lesions that grew after treatment should harbor multiple *RAS* mutations. However, almost all resistant metastatic lesions (except for the RL in patient 1) harbored one acquired *RAS* mutation, as demonstrated by conventional Sanger sequencing and the sensitive PCR-SSO procedure. In the RL in patient 1, multiple *KRAS* mutants were displayed by PCR-rSSO. However, the proportion of mutants was low and was not detected by Sanger sequencing, suggesting the existence of many types of cancer cells. As visualized by computed tomography, the lower proportion of multiple KRAS mutants could explain why the RL continued to shrink with calcification during sequential chemotherapy treatment.

As only a small part of the tumor lesions could be examined, we cannot exclude sampling bias regarding the existence of mutant alleles precluding the assessment of genetic heterogeneity within or among lesions. However, in CRC, *RAS* mutations are believed to occur in the early phase of tumorigenesis, such as developing a small adenoma into a larger adenoma [31]. Thus, an *RAS* mutation spreads homogeneously within the tumor mass, resulting in more than 95% concordance in *RAS* mutation status across different sites of a tumor mass [32]. Therefore, *RAS* mutation-based drug resistance may be attributable to new mutations arising rather than the selection and clonal amplification of an exceedingly small number of pre-existing *RAS* mutant cells. This scenario was also supported by previously reported *in vitro* studies [1,33]. Indeed, Shaffer *et al.* presented that in a *BRAF*-mutant melanoma cell line, the population of resistant cells may arise upon selecting multiple clones that were already present before *BRAF* inhibitor treatment [34]. Of note, these resistant cells arise from profound transcriptional variability at the single-cell level, which involves infrequent, semi-coordinated transcription of several resistance markers at high levels in a small percentage of cells. The addition of the drug then induces epigenetic reprogramming in these cells, converting the transient transcriptional state to a stably resistant state with acquired mutations.

In this study, although we only analyzed four cases and were unable to identify acquired mutations via liquid biopsy before radiographic documentation of PD, we could detect acquired drug resistance-inducing mutations in liquid biopsy collected after radiographical PD by the PCR-rSSO method. Interestingly, patients 1 and 3 experienced radiographic PD during chemotherapy with bevacizumab. Moreover, the blood obtained after confirmation of bevacizumab resistance showed no *RAS* acquired mutations. Moreover, even if acquired mutations occur, they do not always indicate that the drug is ineffective, as demonstrated by the metastatic lesion of the RL in patient 1. Our results strongly emphasize the clinical utility of liquid biopsy in the detection of RAS mutations for decisions regarding anti-EGFR antibody administration to individuals at risk of drug resistance.

## Conclusion

In this study, we intended to establish whether PCR-rSSO can be used to detect acquired mutations in liquid biopsy. Although the sample size is too small to reach significance, our results demonstrated that acquired mutations observed in metastatic lesions with acquired resistance were detected in plasma by the PCR-rSSO method.

**Executive summary**

- We evaluated whether a PCR-reverse sequence-specific oligonucleotide (PCR-rSSO) method can examine the concordance between liquid biopsy and metastatic lesions with acquired resistance.
- Liquid biopsy based on the PCR-rSSO is a successful procedure for capturing acquired mutations with precise information of mutational spectrum that may lend us to reach selective target agents for *RAS* mutations.
- Specimen and liquid biopsy analyses revealed that the patient acquired multiple secondary activating *KRAS* mutations that differed among the metastatic sites following treatment with panitumumab, suggesting a continued process of mutagenesis and the need for alternative treatment strategies in cases treated with anti-EGFR antibodies.
